# ZnO-CeO_2_ Hollow Nanospheres for Selective Determination of Dopamine and Uric Acid

**DOI:** 10.3390/molecules29081786

**Published:** 2024-04-15

**Authors:** Yaru Zhang, Xiaoxia Yan, Yifan Chen, Dongmei Deng, Haibo He, Yunyi Lei, Liqiang Luo

**Affiliations:** 1 Department of Chemistry, Shanghai University, Shanghai 200444, China; zyr0825@shu.edu.cn (Y.Z.); chenyifanchem@shu.edu.cn (Y.C.); hbhe2006@shu.edu.cn (H.H.); shyylei@shu.edu.cn (Y.L.); 2 Department of Physics, Shanghai University, Shanghai 200444, China; dmdeng@shu.edu.cn

**Keywords:** ZnO-CeO_2_, hollow spheres, dopamine detection, uric acid detection, electrochemical sensor

## Abstract

ZnO-CeO_2_ hollow nanospheres have been successfully synthesized via the hard templating method, in which CeO_2_ is used as the support skeleton to avoid ZnO agglomeration. The synthesized ZnO-CeO_2_ hollow nanospheres possess a large electrochemically active area and high electron transfer owing to the high specific surface area and synergistic effect of ZnO and CeO_2_. Due to the above advantages, the resulting ZnO-CeO_2_ hollow spheres display high sensitivities of 1122.86 μA mM^−1^ cm^−2^ and 908.53 μA mM^−1^ cm^−2^ under a neutral environment for the selective detection of dopamine and uric acid. The constructed electrochemical sensor shows excellent selectivity, stability and recovery for the selective analysis of dopamine and uric acid in actual samples. This study provides a valuable strategy for the synthesis of ZnO-CeO_2_ hollow nanospheres via the hard templating method as electrocatalysts for the selective detection of dopamine and uric acid.

## 1. Introduction

Dopamine (DA) and uric acid (UA) are usually co-existing important biological molecules in the human body, playing a crucial role in numerous physiological and pathological features. Some metabolic disturbances in organisms are linked to unnormal levels of DA and UA [1]. Therefore, monitoring DA and UA concentration levels simultaneously is of great significance for analytical and diagnostic applications. DA is a significant neurotransmitter that is closely linked to the regulation of mood and movement [2]. It has a pivotal function in both the cardiovascular and central nervous systems. Abnormal DA level often contributes to the development of serious illnesses like Parkinson’s disease and schizophrenia [3,4]. UA is a significant by-product of purine metabolism and is commonly present in human serum and biological fluids [5]. High concentrations of UA in body fluids could cause myocardial damage, hyperuricaemia and other physiological disorders [6,7]. Consequently, it is vital to develop a convenient and highly efficient approach for the selective detection of DA and UA. Compared to other detection methods such as chromatography, fluorescence and spectrophotometry, the electrochemical method offers the advantages of high sensitivity, quick response times, cost-effectiveness, and convenience, and has aroused more and more attention [8,9,10].

It is well known that DA and UA are simultaneously present in human body fluids, and the oxidation peaks of DA and UA on bare electrodes are very close to each other, resulting in severely overlapping oxidation potentials, which will affect the accurate determination of DA and UA. Therefore, it is extremely essential to explore a kind of suitable modified electrode material to improve the selectivity of DA and UA detection [11]. Electrode materials are the fundamental elements of electrochemical sensors. Transition metal oxides are commonly utilized in sensing and adsorbent materials due to their outstanding physicochemical characteristics, as evidenced by various academic investigations [12,13,14]. ZnO is a transition metal oxide with excellent electrocatalytic properties. Inherent defects such as interstitial zinc atoms and oxygen vacancies have electrical conductivity and electrochemical properties. ZnO possesses favorable characteristics for electrode materials, including chemical and structural stability, high electron density and low cost. These advantages make it a promising option among electrode candidates [15]. For example, Ganesamurthi et al. fabricated NiO-ZnO composite microspheres to detect harmful flavonoids in biological and botanical samples, achieving a minimum detection limit of 11.0 nM [16]. In the study of Myndrul et al., ZnO tetrapods were modified on MXene nanosheets to detect glucose in human sweat [17]. However, ZnO is prone to inadequate dispersion and particle agglomeration, resulting in reduced electrochemical active sites [18,19,20]. To solve this problem, hollow nanomaterials have attracted more and more attention to enable increased specific surface areas and a large number of reaction sites, and thus to lead to the superior electrochemical sensing property of the ZnO-based materials [21,22,23].

In order to obtain uniform and controllable hollow-structured materials, the hard templating method can be considered as a self-assembly process [24]. However, regretfully, pure ZnO hollow materials are still very difficult to synthesize due to its chemical nature [25]. Therefore, CeO_2_ is introduced into the synthesis system for fabricating the ZnO-based hollow nanospheres [24]. CeO_2_ possesses abundant oxygen vacancies and high oxygen mobility [24]. Although CeO_2_ shows unsatisfactory electrocatalytic activity, it could be utilized as an excellent co-catalyst in combination with ZnO to strengthen the electrocatalytic activity [26]. In this strategy, resorcinol-formaldehyde (RF) resin spheres were used as precursors, while CeO_2_ plays a crucial role for supporting ZnO to keep the hollow structure after removing RF resin spheres. Besides the above essential role, CeO_2_ could also expedite electronic transmission between the electrode and the surface-modified electrode material, thus acting as a desirable co-catalyst [27,28,29]. Hence, the amalgamation of transition metal oxides with CeO_2_ renders CeO_2_ a suitable catalyst carrier, exploiting the structural features and synergistic effects between transition metal oxides to enhance the electrochemical efficiency of the sensor. 

In this study, ZnO-CeO_2_ nanospheres with hollow structures were designed and synthesized by combining sol-gel self-assembly and the hard-templating method. Through the accurate regulation of RF resin spheres with ZnO and CeO_2_ precursors, RF@ZnO-CeO_2_ core-shell spheres were formed via the self-assembly process. After calcination at a high temperature, ZnO-CeO_2_ hollow nanospheres were successfully synthesized by removing the template of RF spheres. The as-obtained material was modified on a glassy carbon electrode (GCE) to construct an enzyme-free sensor for the selective determination of DA and UA. As expected, ZnO-CeO_2_/GCE was able to selectively detect DA and UA, and was utilized with success in measuring DA and UA in real samples, demonstrating its promising potential for practical applications.

## 2. Results and Discussion

### 2.1. Morphological Characterization and Synthesis Mechanism of ZnO-CeO_2_ Hollow Nanospheres

**Figure 1:**
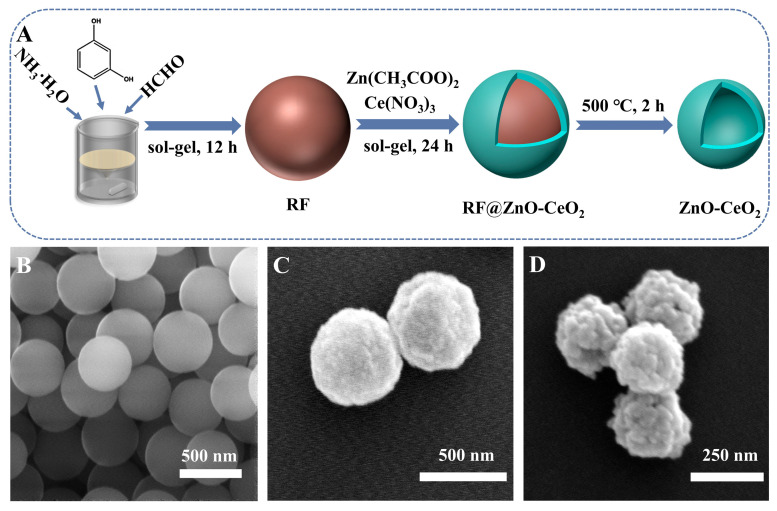
(**A**) Diagrammatic representation of the synthesis of ZnO-CeO_2_ hollow nanospheres. SEM images of RF (**B**), RF@ZnO-CeO_2_ (**C**) and ZnO-CeO_2_ (**D**).

**Figure 2:**
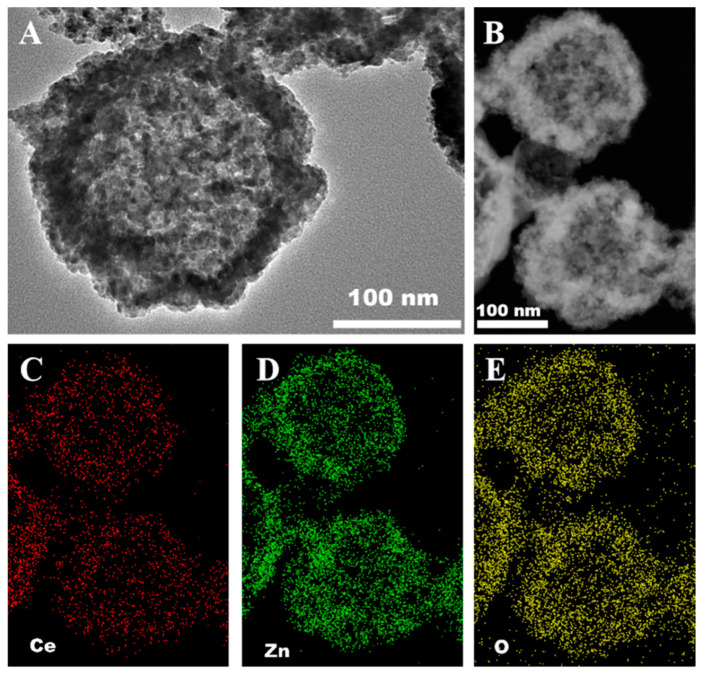
(**A**,**B**) TEM images of ZnO-CeO_2_ hollow spheres. (**C**–**E**) Elemental mapping images of ZnO-CeO_2_.

In this study, ZnO-CeO_2_ composite material with hollow structure was designed and synthesized by hard-templating method. As shown in Figure 1A, the RF template with uniform size was synthesized by resorcinol and HCHO, and the RF@ZnO-CeO_2_ core-shell organic–inorganic complexes were formed by sol–gel and self-assembly deposition of Zn^2+^ and Ce^4+^ species on the RF template surface in certain proportions. ZnO-CeO_2_ hollow nanospheres were formed by removing the template of RF resin spheres. Herein, the morphologies of RF, RF@ZnO-CeO_2_ and ZnO-CeO_2_ were characterized by SEM. Notably, RF spheres were smooth and possessed a consistent size with a distributed diameter of approximately 400 nm (Figure 1B). However, after calcination at 500 °C, the diameters decreased to 200−300 nm and the sphere surface was clearly rougher due to the RF template removing and, further, the ZnO-CeO_2_ layer shrinking (Figure 1C,D). To further observe the internal structure of the ZnO-CeO_2_ spheres in detail, TEM was employed with an element analysis of the ZnO-CeO_2_ layer. As shown in Figure 2A,B, it was evident that a hollow structure appeared in the composite materials when the RF spheres were removed by calcination at a high temperature of 500 °C. EDS elemental mapping (Figure 2C–E) shows that hollow ZnO-CeO_2_ nanospheres exhibit a uniform distribution of the three elements, Ce, Zn and O, which further reveals the successful synthesis of ZnO-CeO_2_ composites with hollow structures.

### 2.2. Structural and Compositional Characterization of ZnO-CeO_2_ Hollow Nanospheres

**Figure 3:**
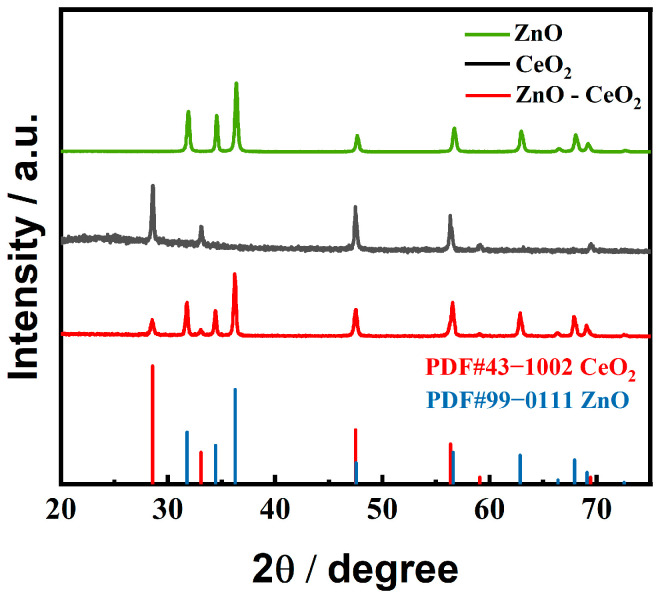
XRD patterns of CeO_2_, ZnO and ZnO-CeO_2_.

Phase composition in ZnO-CeO_2_ hollow nanospheres was further investigated by XRD. As indicated in Figure 3, the XRD patterns provide essential information on the crystal structure of ZnO-CeO_2_ nanospheres. Diffraction peaks were examined at 28.5°, 33.1°, 47.5° and 69.4°, which corresponded to crystal planes of CeO_2_ crystal faces, including (111), (200), (220) and (400) (JCPDS 43-1002). Additionally, diffraction peaks were observed at 31.7°, 34.4°, 36.3°, 47.5°, 56.6°, 62.9°, 66.4°, 67.9°, 69.1° and 72.6°, which correspond to (100), (002), (101), (102), (110), (103), (200), (112), (201) and (004) of ZnO (JCPDS 99-0111) [30,31]. There were no redundant peaks in the XRD pattern of ZnO-CeO_2_ nanospheres, which indicated that the purity of the hollow product was high and well controlled in the synthesis process.

**Figure 4:**
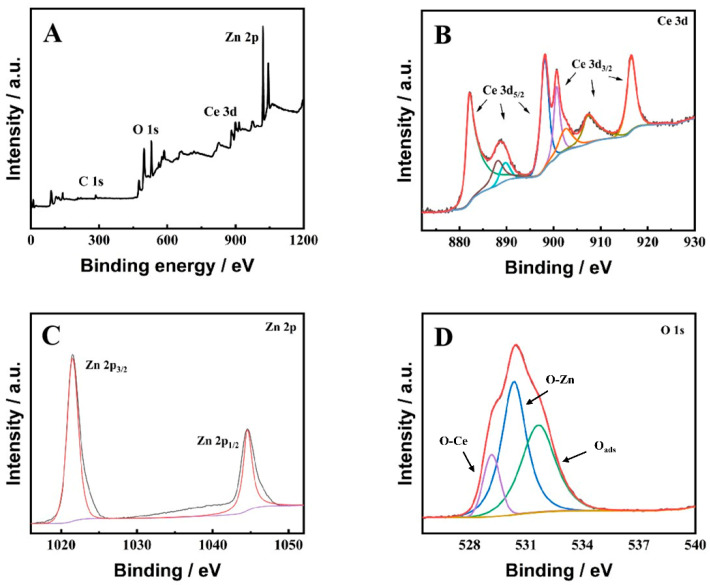
XPS spectra of ZnO-CeO_2_: (**A**) full-width scan; (**B**) Ce 3d; (**C**) Zn 2p; (**D**) O 1s.

The valence states and electron configurations of elements in ZnO-CeO_2_ were further studied by XPS. The full-scan spectra displayed in Figure 4A reveal the presence of four elements in the synthesized functional composites: Zn, Ce, C and O. This finding corroborated the EDS test results. Additionally, Figure 4B displays the Ce 3d spectrum. The spectrum of Ce 3d exhibited eight discernible peaks, in which the peaks at 901.1, 907.8 and 916.7 eV were associated with 3d_3/2_ of Ce^4+^, and the peaks at 882.6, 888.8 and 898.6 eV were associated with 3d_5/2_ of Ce^4+^. Furthermore, it was evident that the composite contains a Ce^4+^/Ce^3+^ redox electric pair, as indicated by two distinctive peaks at 903.1 and 884.9 eV, which originated from Ce^3+^ [31,32,33]. The high-resolution XPS spectrum of Zn 2p (Figure 4C) shows two characteristic peaks at 1021.6 and 1044.6 eV, which could be related to the Zn 2p_3/2_ and Zn 2p_1/2_ orbits of Zn^2+^ [34]. The spectrum for O 1s is presented in Figure 4D, with the O 1s peaks fitted from three peaks at 532.4, 530.8 and 529.8 eV. The O present in the lattice, originating from ZnO and CeO_2_, was responsible for the peaks at 530.8 and 529.8 eV, and the adsorption of O_2_ on the surface of the material was accounted for by the peak at 532.4 eV [27]. The results of XPS further suggested that ZnO-CeO_2_ composite hollow nanospheres were successfully synthesized.

Nitrogen adsorption/desorption isotherms were carried out to further assess the specific surface area of ZnO-CeO_2_ hollow nanospheres. As shown in Figure S1A, the ZnO-CeO_2_ hollow spheres were demonstrated to own a type IV isotherm. ZnO-CeO_2_ hollow nanospheres had a specific surface area of roughly 45.46 m^2^ g^−1^. Compared with ZnO (10.51 m^2^ g^−1^) and CeO_2_ (30.59 m^2^ g^−1^), ZnO-CeO_2_ hollow nanospheres showed increased specific surface area. The higher specific surface area of the spheres mean that more reactive active sites could be provided in the electrochemical process, implying a boosted charge transfer velocity and an improved electrochemical oxidation performance.

### 2.3. Electrochemical Performance of ZnO-CeO_2_ Hollow Nanospheres

**Figure 5:**
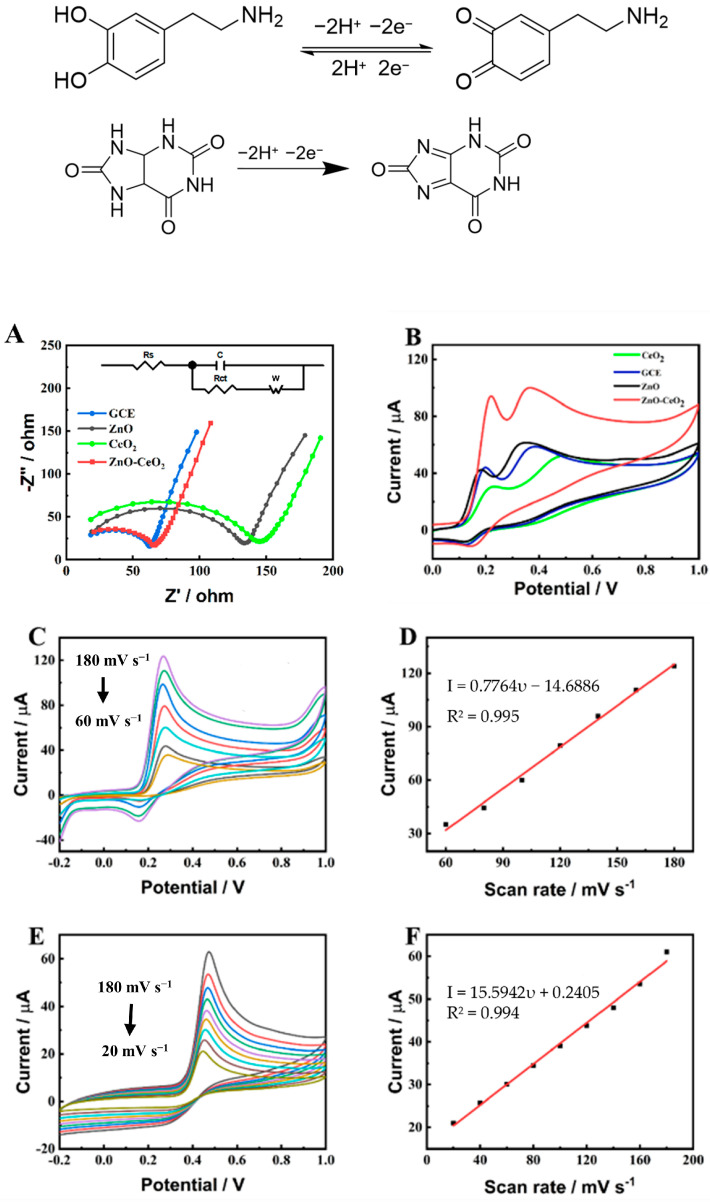
(**A**) EIS spectra recorded in 0.1 M KCl containing 5.0 mM [Fe(CN)_6_]^3–/4–^ at the GCE, ZnO/GCE, CeO_2_/GCE and ZnO-CeO_2_/GCE; (**B**) CV curves of the GCE, ZnO/GCE, CeO_2_/GCE and ZnO-CeO_2_/GCE electrodes in the presence of 2 mM DA and UA; (**C**) CV curves of ZnO-CeO_2_/GCE in 0.1 M PBS containing 2 mM DA at different scan rates (from 60 to 180 mV s^−1^); (**D**) The calibration curve of the linear relationship between the current (I_pa_) and the scan rate (υ); (**E**) CV curves of ZnO-CeO_2_/GCE in 0.1 M PBS containing 2 mM UA at different scan rates (from 20 to 180 mV s^−1^); (**F**) The calibration curve of the linear relationship between the current (I_pa_) and the scan rate (υ).

In order to evaluate the hollow ZnO-CeO_2_ modified electrode’s capacity for charge transfer, electrochemical impedance spectroscopy (EIS) was employed, as illustrated in Figure 5 [35]. The surface characteristics of the modified electrode were examined by using [Fe(CN)_6_]^3−/4−^ as redox probe for analyzing the charge transfer capacity of the electrode. As shown in Figure 5A, compared with ZnO and CeO_2_, the ZnO-CeO_2_/GCE shows a lower R_ct_ value. It is proved that the ZnO-CeO_2_ hollow nanospheres exhibit a more excellent electron transfer property due to the high specific area and great synergistic effect between ZnO and CeO_2_. The EIS of the GCE shows a lower R_ct_ value compared with the ZnO-CeO_2_/GCE electrode. This is because nafion was used for fixing ZnO-CeO_2_ hollow nanospheres on GCE, which would block the diffusion of [Fe(CN)_6_]^3−/4−^ and increase the R_ct_ value of the ZnO-CeO_2_/GCE electrode [35,36]. CeO_2_, as a co-catalyst, can accelerate the electron transfer among ZnO-CeO_2_/GCE due to abundant oxygen vacancies of CeO_2_. As shown in Figure 5B, compared with the pure ZnO and CeO_2_, ZnO-CeO_2_ hollow nanospheres exhibit the highest oxidation peak at 0.25 V and 0.4 V, respectively. This is in accordance with the EIS results in Figure 5A, suggesting that the synergistic effect of CeO_2_ and ZnO could promote the charge transfer, enhance the conductivity and then improve the electrocatalytic activity of ZnO-CeO_2_ hollow nanospheres. In this research, the effect of different scanning rates on the detection of DA and UA by the ZnO-CeO_2_/GCE electrode was tested by cyclic voltammetry in 0.1 M PBS (pH 7.0). As seen in Figure 5C,E, the peak current of DA and UA increased progressively as the scanning rate increased. Within a specific range, the peak current was proportional to the scanning rate. The peak current of DA (Figure 5D) was related to the scanning rate, I = 0.7764υ − 14.6886 (R^2^ = 0.995). The oxidation peak current of UA (Figure 5F) was directly proportional to the scanning rate, I = 15.5942υ + 0.2405 (R^2^ = 0.994). According to the aforementioned findings, the surface adsorption controlled the electrochemical responses between DA and UA on the ZnO-CeO_2_/GCE electrode. The electro-oxidation reactions of DA and UA on ZnO-CeO_2_/GCE are a two-electron-transfer process, and the reaction mechanisms can be expressed as follows [37]:

### 2.4. Determination of DA and UA on ZnO-CeO_2_/GCE

**Figure 6:**
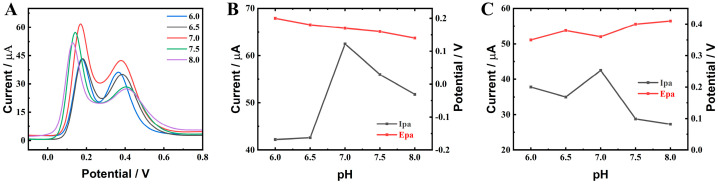
Performance of ZnO-CeO_2_/GCE for the detection of DA and UA under different pH conditions (**A**). pH against oxidation peak current (Ipa) and oxidation peak potential (Epa) of DA (**B**) and UA (**C**).

In order to enable the ZnO-CeO_2_/GCE electrode to detect DA and UA under optimal experimental conditions, the material ratio of Ce^2+^:Zn^2+^, electrolyte concentration and modifier concentration were optimized by DPV. Figure S2A,B show the effect of the material ratio on the electrochemical sensor performance. When the material ratio was Ce^2+^:Zn^2+^ = 1:4, the oxidation peak currents of DA and UA were the highest, so 1:4 was chosen as the best material ratio. Figure S3A,B display the effect of the concentration of the modifier on the performance of the electrochemical sensor. By comprehensive comparison, the oxidation peak currents of DA and UA were the greatest at 4.0 mg mL^−1^, so 4.0 mg mL^−1^ was selected to be the ideal concentration of modifier load. As shown in Figure 6B,C, the peak current gradually increased when the pH of the PBS solution increased from 6.0 to 7.0. However, when it increased to 8.0, the electrochemical signal gradually decreased. Therefore, a pH 7.0 of 0.1 M was selected for the determination of DA and UA.

**Figure 7:**
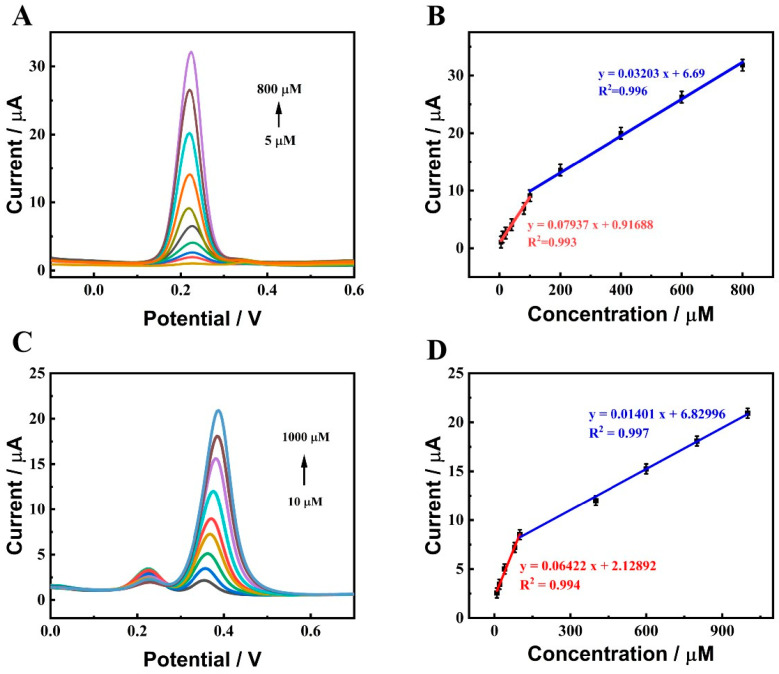
(**A**,**C**) DPV responses of ZnO-CeO_2_/GCE with concentrations of DA and UA in supporting electrolyte solution; (**B**,**D**) Corresponding calibration curve.

**Figure 8:**
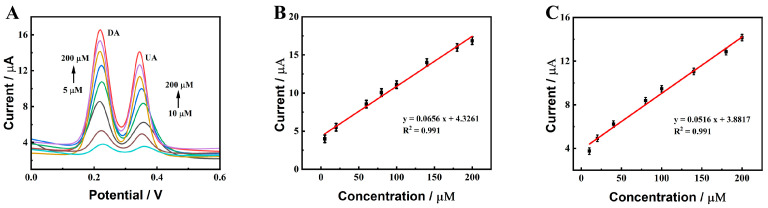
(**A**) DPV responses of ZnO-CeO_2_/GCE with different concentrations of DA and UA in supporting electrolyte solution; (**B**,**C**) Corresponding calibration curves.

Under the optimal experimental parameters, the DPVs of DA and UA with different concentrations were measured at ZnO-CeO_2_/GCE. First, the concentration of UA was retained at 10 μM, and the concentrations of DA increased. As indicated in Figure 7A, in 0.1 M PBS (pH 7.0), the oxidation peak current increased following the change in DA concentration. Figure 7B shows the linear fitting curve between the peak current and DA content. It was displayed that the peak current and DA concentration in the 5–100 μM had a good linear relationship. The linear equation could be fitted to I (μA) = 0.0794C (μM) + 0.9169 (R^2^ = 0.993) and a sensitivity of 1122.8 μA mM^−1^ cm^−2^. In the range of 100–800 μM, the linear correlation between the DA concentration and the peak current was good. The linear equation could be fitted as I (μA) = 0.0320 C (μM) + 6.690 (R^2^ = 0.996) and the sensitivity was calculated as 452.99 μA mM^−1^ cm^−2^. Subsequently, the content of DA was fixed at 20 μM and the concentration of UA increased. As shown in Figure 7C, in 0.1 M PBS (pH 7.0) solution, the oxidation peak current changed with the increase in UA concentration. Figure 7D shows the linear fitting curve between the oxidation peak current and UA concentration. It was seen that the oxidation peak current and UA concentration in the 10–100 μM had a good linear connection. The linear equation could be fitted as I (μA) = 0.0642C (μM) + 2.1289 (R^2^ = 0.994) and the sensitivity was 908.53 μA mM^−1^ cm^−2^. The linear relationship between the oxidation peak current and UA content was well established in the range of 100–1000 μM. The linear equation was fitted as I (μA) = 0.0140C (μM) + 6.8299 (R^2^ = 0.997) and the sensitivity was calculated as 198.20 μA mM^−1^ cm^−2^. To the best of our knowledge, in the lower concentration region, product molecules on the electrode surface are easier to desorb than in the higher concentration region, which is beneficial for obtaining more rapid electrochemical reactions and a more sensitive response for the electrocatalysis of DA and UA [38]. Therefore, two linear dynamic ranges occur at two different concentration regions. The detection limits were 0.39 μM for DA and 0.49 μM for UA (S/N = 3), separately, indicating that the constructed ZnO-CeO_2_/GCE had good sensing performance in the detection of DA and UA. Figure 8 shows that the peak current increases as the analyte concentration increases. The well-defined peak potentials for DA and UA are +0.230 V and +0.386 V, respectively. The spacing between the two peaks is 0.156 V, which is sufficient for the simultaneous detection of DA and UA.

By comparing the linear range and detection limits of other reported DA and UA sensors (Table 1), the prepared ZnO-CeO_2_/GCE sensor can meet the requirements for the selective determination of DA and UA in real samples, and has great practical application potential.

In order to explore the selectivity of the ZnO-CeO_2_/GCE electrochemical sensor, some common compounds in human serum: ascorbic acid, urea, glucose, K^+^, Na^+^, Cl^−^ and Trp were tested by DPV. As illustrated in Figure S4A, the addition of interferences had little impact on the intensity of electrical signals generated by DA and UA, and the relative current value changed no more than 0.94%, proving that the ZnO-CeO_2_/GCE electrode had a strong anti-interference ability. One electrode was used to measure DA and UA seven times to assess repeatability. The results showed that ZnO-CeO_2_/GCE had good repeatability (Figure S4B). In addition, seven ZnO-CeO_2_/GCE electrodes were prepared in the same way, and the reproducibility of the ZnO-CeO_2_/GCE electrodes was assessed by the responding current to 250 μM DA and UA (Figure S4C). The results showed that ZnO-CeO_2_/GCE had good reproducibility. To further assess the stability of the sensor, ZnO-CeO_2_/GCE was stored for 15 days, and the peak currents of the electrode to 250 μM DA and UA were recorded every day. After 15 days, the peak current remained 93.8% of the incipient current. The above test results showed that ZnO-CeO_2_/GCE exhibited excellent anti-interference, repeatability, reproducibility and stability.

The performance of the constructed ZnO-CeO_2_/GCE in the detection of DA and UA in real samples was further evaluated. The concentrations of DA and UA in human serum were, respectively, determined by the ZnO-CeO_2_/GCE electrochemical sensor. In this study, the standard addition method was used to test DA and UA in human serum. The blood samples were first preliminarily pre-treated and the supernatant was obtained by centrifugation at 10,000 rpm for 10 min and diluted 50 times with 0.1 M PBS (pH 7.0). Then, the DA and UA standard solutions were added. DA concentrations of 10 μM, 20 μM and 30 μM and UA concentrations of 20 μM, 30 μM and 40 μM were added to human serum, respectively, and the concentrations of DA and UA were obtained by the standard addition method and the aforementioned calibration curve. As listed in Table 2 and Table 3, the results show that the prepared ZnO-CeO_2_/GCE sensors met the requirements for detecting DA and UA in practical applications.

## 3. Experimental

### 3.1. Materials

Zn(CH_3_COO)_2_·4H_2_O, Ce(NO_3_)_3_·6H_2_O, anhydrous ethanol, ammonia water (NH_3_∙H_2_O, 28%), resorcinol, formaldehyde (HCHO, 37%), glucose (Glu), KH_2_PO_4_, K_2_HPO_4_, KCl and NaCl were provided by Sinopharm Chemical Reagent Co., Ltd. (China). Tryptophan (Trp) and ascorbic acid (AA) were purchased from Sigma (USA). Dopamine (DA), uric acid (UA) and urea (CH_4_N_2_O) were acquired from Alfa Aesar. All chemicals were of analytical grade. All the water utilized in the experiments was ultrapure water (18.25 MΩ cm).

### 3.2. Instruments

The morphologies and dimensions were examined using scanning electron microscopy (SEM, Phenom·Pharos·G1, Phenom, Eindhoven, The Netherlands). Energy-dispersive spectroscopy (EDS) images were collected from transmission electron microscopy (TEM, JEM-2100F, JEOL LTD, Tokyo, Japan). The crystal structures were analyzed by X-ray diffraction (XRD, D/MAX 2500, Rigaku, Tokyo, Japan), working with Cu Kα radiation (λ = 1.5418 Å, 0.08° s^−1^). Analysis of element composition and valence structure on X-ray photoelectron spectroscopy (XPS, Escalab 250 XI, Thermo Scientific, Waltham, MA, USA). The N_2_ adsorption–desorption isotherms were determined with an Automatic adsorption instrument (ASAP 2460, Micromeritics, Norcross, GA, USA). To compute the specific surface area, the Brunauer–Emmett–Teller (BET) equation was utilized. Before the test, the samples were preprocessed under a vacuum for 6 h at 100 °C. All electrochemical performance tests were finished on an electrochemical workstation (CHI 660e, CH Instruments, China) equipped with the three-electrode system. 

### 3.3. Synthesis of RF Resin Spheres

The synthesis method of RF resin balls was improved by the reported method [45]. An amount of 300 µL NH_3_·H_2_O was added to 16 mL CH_3_CH_2_OH and 40 mL deionized water. Then, 0.1 g resorcinol and 280 µL HCHO were added and stirred overnight. The product was subjected to centrifugation, washed and dried. These procedures yielded powdered microspheres of RF.

### 3.4. Synthesis of ZnO-CeO_2_ Hollow Nanospheres

Utilizing the sol-gel concept and the hard-templating approach, ZnO-CeO_2_ hollow nanospheres based on RF nanospheres were created. In the typical synthesis, Zn(CH_3_COO)_2_ and Ce(NO_3_)_3_ were chosen as precursors of ZnO and CeO_2_ composite with a proper molar ratio of 4:1. First, 0.05 g RF was added to 8 mL anhydrous ethanol and 2 mL acetonitrile. After sonicating for 30 min, we slowly added 2 mM Zn(CH_3_COO)_2_·4H_2_O and Ce(NO_3_)_3_·6H_2_O ethanol solution to 5 mL. We stirred the reaction for 24 h, followed by washing through centrifugation and drying in a vacuum drying oven at 60 °C to obtain RF@ZnO-CeO_2_. Finally, the RF@ZnO-CeO_2_ composites were heated at 500 °C for 2 h to obtain the final sample, ZnO-CeO_2_. The synthesis of ZnO and CeO_2_ nanospheres followed the same procedure.

### 3.5. Fabrication of the Modified Electrode

The electrode surface of GCE was cleaned with 0.1 and 0.05 μm aluminum powder; any impurities were then washed away with deionized water. Then, 5 μL ZnO-CeO_2_ (5 mg mL^−1^) were uniformly dispersed on the GCE and placed under the infrared light to dry. ZnO-CeO_2_/GCE were obtained for detecting DA and UA.

## 4. Conclusions

In this study, ZnO-CeO_2_ composite nanospheres with hollow structures were successfully fabricated via the hard templating method. The as-obtained ZnO-CeO_2_ hollow nanospheres possess high specific surface areas and abundant active sites, which is favorable for electron transfer and electrocatalysis. Taking advantage of the synergistic effect between ZnO and CeO_2_, the synthesized ZnO-CeO_2_ hollow nanospheres exhibit superior electrocatalytic activities to the oxidations of DA and UA in 0.1 M PBS (pH 7.0) solution. The developed sensor displays wide linear ranges (5–800 μM for DA, and 10–1000 μΜ for UA), high sensitivities (1122.86 μA mM^−1^ cm^−2^ for DA, and 908.53 μA mM^−1^ cm^−2^ for UA) and low detection limits (0.39 μM for DA, and 0.49 μM for UA). In addition, the fabricated ZnO-CeO_2_/GCE was also successfully utilized to measure the concentration levels of DA and UA in human serum samples. This work proves that ZnO-CeO_2_ hollow nanospheres could be applied for selectively detecting DA and UA. 

## Figures and Tables

**Table 1:** Performance comparison of ZnO-CeO_2_/GCE with other DA and UA sensors.

Modified Material	Linear Range (μM)		Detection Limit (μM)		Reference
	DA	UA	DA	UA	
CuNP ^a^ @rGO ^b^	20–1000	60–900	2.26	6.72	[39]
N-doped carbon Supported iron	5–900	4–300	3.21	3.348	[40]
HNGA ^c^	0.6–75	0.4–50	0.22	0.12	[41]
HNAC ^d^	2–10	20–100	0.401	2.800	[42]
Fe_3_O_4_	10–100	20–160	4.5	14	[43]
PPy ^e^ -Co-NNC ^f^	1–50	2–500	0.025	0.411	[44]
PMo_12_ ^g^ @MIL-100(Fe) ^h^ @PVP ^i^	1–247	5–406	0.586	0.372	[37]
ZnO-CeO_2_	5–800	10–1000	0.39	0.49	This work

Notes: ^a^ nanoparticles, ^b^ reduced graphene oxide, ^c^ holey nitrogen-doped graphene aero gel, ^d^ homogeneous nanoparticles distributed on amorphous carbon, ^e^ Polypyrrole, ^f^ cobalt single-atom nanozymes of tubular bis-paraben nitrogen–carbon, ^g^ PMo_12_O_40_^3−^, ^h^ C_9_H_5_FeO_7_, ^i^ polyvinylpyrrolidone.

**Table 2:** Determination of DA in human serum.

Serum Sample	Added (μM)	Found (μM)	Recovery (%)	RSD (%)
1	10	9.89	98.9	0.96
2	20	19.94	99.7	1.26
3	30	30.4	101.3	1.49

**Table 3:** Determination of UA in human serum.

Serum Sample	Added (μM)	Found (μM)	Recovery (%)	RSD (%)
1	20	19.57	97.9	1.29
2	30	30.45	101.5	1.23
3	40	40.2	100.5	1.47

## Data Availability

Data are contained within the article and Supplementary Materials.

## Supplementary Materials

The following supporting information can be downloaded at: https://www.mdpi.com/article/10.3390/molecules29081786/s1, Figure S1: (A) Nitrogen adsorption/desorption isotherm (B) and pore size distribution of ZnO-CeO_2_ hollow spheres; Figure S2: The plot between the various molar ratios of Ce and Zn against oxidation peak current (Ipa) and oxidation peak potential (Epa) of DA(A) and UA(B); Figure S3: Surface modification material concentration against oxidation peak current (Ipa) and oxidation peak potential (Epa) of DA(A) and UA(B); Figure S4: (A) Current response of ZnO-CeO_2_/GCE to 200μM DA, UA and 1 mM interferences; (B) The repeatability of ZnO-CeO_2_/GCE electrodes to DA and UA sensing; (C) The reproducibility of ZnO-CeO_2_/GCE electrodes to DA and UA sensing; (D)Long-time stability of ZnO-CeO_2_/GCE.
