# Supplementary material for: ZnO-CeO2 Hollow Nanospheres for Selective Determination of Dopamine and Uric Acid
Source: Molecules. 2024 Apr 15;29(8):1786. doi: 10.3390/molecules29081786 (PMC11051899; doi:10.3390/molecules29081786)
Supplement: Supplementary file 1 [file molecules-29-01786-s001.zip › molecules-2945344-supplementary.pdf]

Supporting Information

ZnO-CeO_2 Hollow Nanospheres for Selective Determination of Dopamine and Uric Acid

Yaru Zhang [1], Xiaoxia Yan [1,*], Yifan Chen [1], Dongmei Deng [2], Haibo He [1], Yunyi Lei [1] and Liqiang Luo [1,*]

1 Department of Chemistry, Shanghai University, Shanghai 200444, China; zyr0825@shu.edu.cn (Y.Z.); chenyifanchem@shu.edu.cn (Y.C.); hbhe2006@shu.edu.cn (H.H.); shyylei@shu.edu.cn (Y.L.)

2 Department of Physics, Shanghai University, Shanghai 200444, China; dmdeng@shu.edu.cn

* Correspondence: xxyan@shu.edu.cn (X. Yan); luck@shu.edu.cn (L. Luo); Tel.: +86 21 66132404

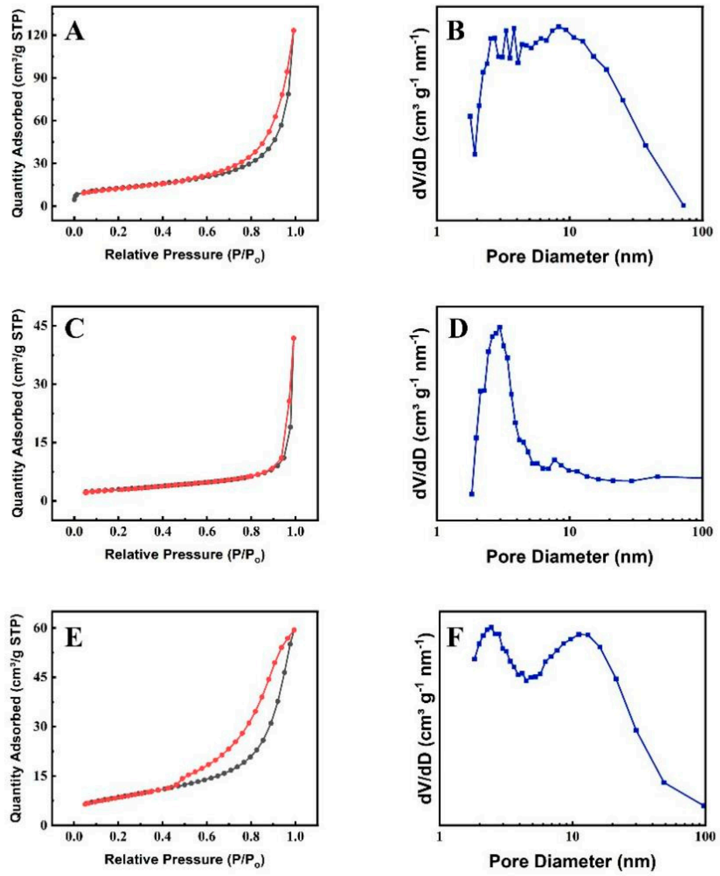

Figure S1 Nitrogen adsorption/desorption isotherm of ZnO-CeO_2 hollow spheres (A), ZnO (C) and CeO_2 (E). Pore size distribution of ZnO-CeO_2 hollow spheres(B), ZnO (D) and CeO_2 (F).

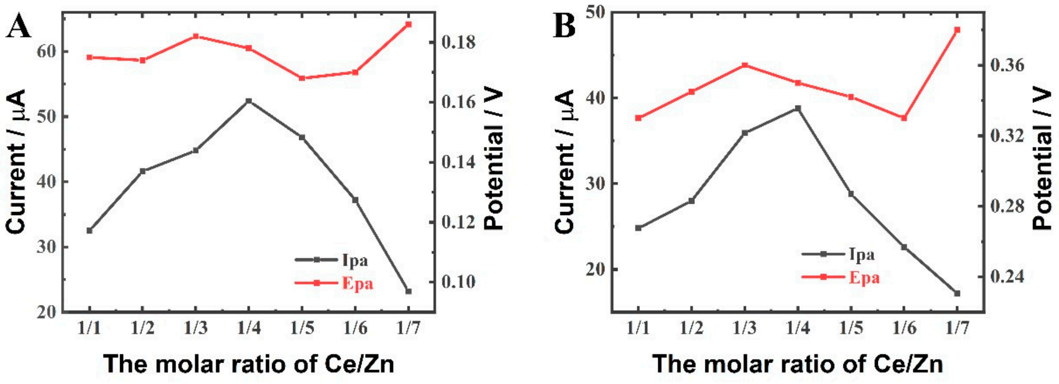

Figure S2 The plot between the various molar ratios of Ce and Zn against oxidation peak current (Ipa) and oxidation peak potential (Epa) of DA(A) and UA(B).

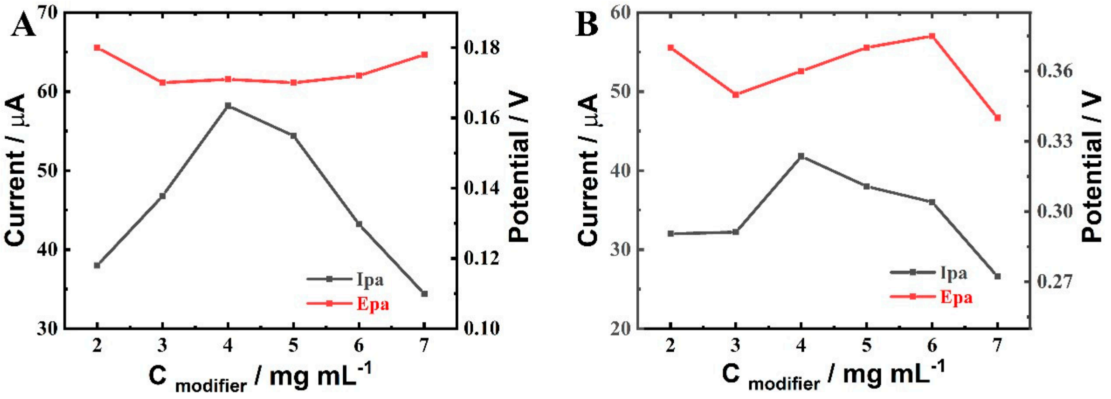

Figure S3 Surface modification material concentration against oxidation peak current (Ipa) and oxidation peak potential (Epa) of DA(A) and UA(B).

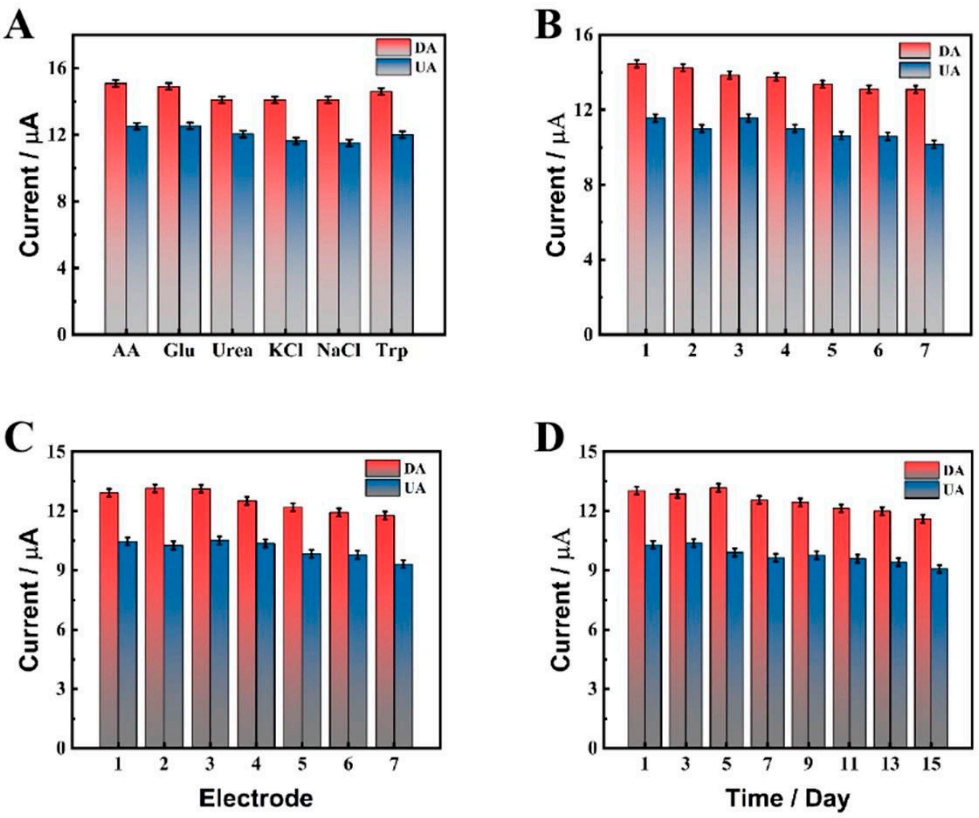

Figure S4 (A) Current response of ZnO-CeO2/GCE to 200μM dopamine, uric acid and 1 mM interferences; (B) The repeatability of ZnO-CeO2/GCE electrodes to DA and UA sensing; (C) The reproducibility of ZnO-CeO2/GCE electrodes to DA and UA sensing; (D)Long-time stability of ZnO-CeO2/GCE.
